# The relationship between executive function and the association of motor coordination difficulties and social communication deficits in autistic children

**DOI:** 10.3389/fpsyt.2024.1363406

**Published:** 2024-03-26

**Authors:** Tingfeng Gu, Chengkai Jin, Lizi Lin, Xin Wang, Xiuhong Li, Jin Jing, Muqing Cao

**Affiliations:** ^1^ Maternal and Child Health Department, School of Public Health, Sun Yat-sen University, Guangzhou, China; ^2^ Guangdong Provincial Engineering Technology Research Center of Environmental Pollution and Health Risk Assessment, Department of Occupational and Environmental Health, School of Public Health, Sun Yat-sen University, Guangzhou, China; ^3^ School of Sport and Health, Guangzhou Sport University, Guangzhou, China

**Keywords:** autism spectrum disorders, motor coordination, social communication deficits, executive function, mediating effect

## Abstract

**Background:**

Motor coordination difficulties could contribute to social communication deficits in autistic children. However, the exploration of the mechanism implicated in these claims has been limited by the lack of potential confounders such as executive function (EF).

**Methods:**

We investigated the role that EF plays in the relationship between motor coordination and social communication in a school-aged autistic population via a structural model in a statistically robust manner. The results of questionnaires, including the Developmental Coordination Disorder questionnaire, the Behavior Rating Inventory of Executive Function, and the Social Responsiveness Scale, were collected to measure motor coordination, social communication deficits, and EF.

**Results:**

A total of 182 autistic children (7.61±1.31 years, 87.9% boys) were included in the final analysis. In the model with EF as a mediator, the total effect (*β*=-0.599, *P*<0.001) and the direct effect (*β*=-0.331, *P* =0.003) of motor coordination function on social communication were both significant among autistic children without intellectual disability (ID), as were indirect effects through EF (*β*=-0.268, *P*<0.001).

**Conclusion:**

EF partially mediates the motor coordination and social communication correlation among autistic children. We suggest that motor coordination should be included in the routine evaluation of autistic surveillance and rehabilitation procedures.

## Introduction

Autism Spectrum Disorder (ASD) is an increasingly prevalent neurodevelopmental disorder with 1 in 44 children diagnosed with ASD (1). As one of the core symptoms of autistic children, social communication deficit is challenging for autistic children in daily life and negatively impacts their prognosis (2, 3). Additionally, it was reported that 86.9% of autistic children were at risk for motor coordination difficulties (4), and correlational studies supported the positive relationship between motor coordination function and social communication in this population (5, 6). Recent evidence also suggested that motor-related interventions improved social communication in autistic children (7, 8), highlighting the potential importance of motor coordination function in understanding the etiology of autistic children.

Although a review of the literature showed a close relationship between motor coordination function and the social communication of autistic children (see **Supplementary Table 1**), the nature of the association remains unclear. Taverna et al. proposed a bidirectional model: autistic children who had better motor coordination function responded more positively to interventions and benefited more in terms of alleviating core symptoms, including social communication. Inversely, improvement in social communication might lead children to engage more in physical activities, thus consequently displaying significant improvement in the motor domain (9). This model was partially supported by several studies of motor-related intervention among autistic children (7). Therefore, to better understand the mechanism underlying the social communication deficit of autistic children and to support early intervention practices for educators, it is urgent to explore the relationship between motor coordination and social communication and the potential mediators.

Autistic children with motor coordination difficulties usually exhibit poor performance in gross and fine motor, coordination, postural control, standing balance, etc. (10); these essential motor skills are usually conducted in a sequence of movements that involves aspects of executive function (EF) (11). EF is a set of higher-order cognitive processes that regulate, monitor and control cognition, emotions, and behavior. Evidence also suggested that motor coordination difficulties were associated with deficits of EF (12), and the improvement of EF could benefit from motor-related interventions (13). Meanwhile, better EF could also predict superior social communication functions among autistic children (14–16), possibly because EF improved social communication by promoting higher-order processes, such as emotional and cognitive regulation (17). Additionally, EF-related intervention also supported this association (18). Thus, EF may act as a potential mediator in the relationship between motor coordination difficulties and social communication deficits.

In **Supplementary Table 1**, we listed the previous studies covering motor coordination, EF, and social communication in autistic children. Most studies only explored the link between motor coordination and social communication; however, the results remained inconclusive. Two studies identified no significant differences in the relationship (19, 20). A limited sample size (21–27), a wide age range among participants (5, 19, 20, 28–33), and the use of various measurement tools for assessing motor coordination and social communication (20, 34, 35) might compromise the generalizability of the current findings. In terms of EF, most studies have considered the association between motor coordination function and EF or between EF and social communication separately. Only one study on intervention research for EF exists, indicating that motor-related interventions may improve both social communication function and EF (36). Moreover, it is worth noting that the indirect effect of EF difficulties on the relationship between motor coordination difficulties and internalizing symptoms was once discussed (37), highlighting the necessity to explore the potential effect of EF on motor difficulties and social communication among autistic children. Furthermore, there was evidence that autistic children with intellectual disability (ID) were distinctly different from those with normal cognition in respect of both motor coordination and social communication (31). Thus, it is necessary to take cognitive levels into consideration when determining their potential impact on this relationship.

This research aimed to gain an understanding of the association between motor coordination difficulties, EF, and social communication deficit among autistic children aged 6-12 years and explore the potential role of EF in the motor coordination–social communication correlation. It was observed that low-functioning autistic children faced challenges in completing the performance-based measurements (38). As a result, the standardization of the tests and the reliability of the results were compromised. Additionally, informant-report measures, completed by parents who have observed the child over a substantial period, offer higher ecological validity compared to lab-based measures (39). These measures effectively capture behaviors in “real-world” settings, providing valuable insights into the challenges that autistic children face in their daily lives (17). Thus, in this research, a total of 118 autistic children were recruited, and motor coordination function, EF, and social communication were evaluated by the validated parent-reported questionnaires or observational scales. Structural equation modeling (SEM) was employed to examine the relation. We hypothesize that 1) social communication deficits could be positively predicted by motor coordination difficulties among autistic children, and 2) EF, as the mediator in this relationship, may be affected by motor coordination difficulties and further impair social communication. To the best of our knowledge, this is the first study to explore the potential role of EF in the relationship between motor coordination difficulties and social communication. Additionally, we relied on child psychologists/psychiatrists to diagnose autistic children, and autistic children with and without ID were analyzed separately.

## Materials and methods

### Participants and data collection procedure

The baseline data of autistic children from an ongoing study “the Guangzhou Longitudinal Study of Children with ASD” in China were used. All the samples in this study were recruited from March 10, 2019, to September 7, 2022, from the Research Center of Children and Adolescent Psychological and Behavioral Development in the Department of Public Health, Sun Yat-sen University. The children had been diagnosed with autistic children by hospitals at the beginning of the recruitment period, and the diagnosis was confirmed by a child psychiatrist on the research team using the Diagnostic and Statistical Manual of Mental Disorders, Fifth-Revision (DSM-5) criteria (40), and the Childhood Autism Rating Scale (CARS) (41), to obtain a reliable autistic sample. The additional inclusion criteria included (1) aged from 6 years 0 months to 12 years 11 months; (2) the voluntary participation of the children’s parents; (3) no other neurodevelopmental disorders; and (4) no severe sensory, perceptual disorders or physical handicaps. To ensure the independence of the observations, if a family had two or more eligible children, only the first-born child was recruited. A total of 182 autistic children were enrolled and comprised those who were able to complete all the assessments. Parent-reported questionnaires were used to obtain demographic information, motor coordination function, EF, and social function. A licensed researcher conducted a behavior evaluation of the child, which included the use of the CARS and the Wechsler Intelligence Scale for Children (Fourth version, WISC-IV, Chinese version) (42). This study was approved by the Ethics Committee of the author’s institution, and signed written informed consent was obtained before the questionnaire was completed.

### Motor coordination assessment

The Developmental Coordination Disorder Questionnaire (DCDQ, Chinese version) (43) was used to measure motor coordination. The DCDQ is a 17-item parent-reported questionnaire consisting of three domains of motor coordination which were fine motor/handwriting, general coordination, and control during movement. The total score ranges from 17 to 85, with higher scores associated with better motor coordination, as well as its subscales. The Cronbach’s alpha value of the total DCDQ score in this study is 0.700. DCDQ is a reliable instrument for screening children at risk for motor coordination deficits, including autistic children (44).

### Executive function assessment

The Behavior Rating Inventory of Executive Function (BRIEF, Chinese version) (45) was used to assess the EF, which is used extensively on autistic children and adolescents (17, 46). The BRIEF is an 86-item parent-reported scale that can be organized into two composite indices: the behavioral regulation index (BRI) and metacognitive index (MCI). The BRI was derived from the inhibit, shift/flexibility, and emotional control subscale, and the MCI was derived from the initiate, working memory, plan/organize, organization of materials, and monitor subscales. Then, a standardized total score was generated from both of the indices to account for the EF of children, with higher scores related to increased EF difficulties.

### Social communication function assessment

The Social Responsiveness Scale (SRS, Chinese version) (47) was used to evaluate the children’s social function. The SRS is a 65-item parent-reported questionnaire consisting of five domains (i.e., social awareness, social cognition, social communication, social motivation, and autistic mannerisms), which is a widely used rating form for measuring the degree of social communication deficits in autistic children (48, 49). For each question, the parents were asked to describe their child’s behavior over the last six months. Differed from other tools, SRS provides a continuous assessment (from impaired to above average) of social communication function rather than a categorical (yes/no) one. The total possible scores range from 0 to 195, with higher scores indicating increased social impairment. The Cronbach’s alpha value of the total SRS score in this study is 0.879.

### Covariates assessment

The information gathered from the children’s caregivers included their sociodemographic characteristics and children’s history. The factors examined were age, gender, if the participant is an only child in the family, handedness, caregiver of the child, maternal age, maternal education level, household income, and intervention history of ASD. These factors were considered potential covariates due to reported associations in previous studies with various aspects of development, including motor, executive and social function (5, 34, 50).

### Cognition assessment

The Wechsler Intelligence Scale for Children (Fourth version, WISC-IV, Chinese version) (42) was used to identify the global intellectual functioning as full-scale IQ (FSIQ). According to the DSM-5, children with an FSIQ < 70 were classified into the ASD-ID group and others were classified into the ASD-only group.

### Statistical analysis

Data analyses were conducted during March and April 2023. The statistical analyses were performed using the Statistical Program for Social Sciences (SPSS) version 25.0 and Mplus version 8 (Los Angeles, CA) software packages. Continuous variables and categorical variables are presented as either mean (SD) values or percentages, respectively.

The intercorrelations of DCDQ, EF, and SRS were explored using Pearson correlations. To test the relationships between DCDQ, EF, and SRS, the structural equation modeling technique (SEM) was employed using the maximum likelihood robust (MLR) method in Mplus. MLR is a method widely used to ensure better performance when using nonnormal data, especially in studies with a small sample size (N<400) (51). In SEM, the proposed hypothesized model is evaluated for the goodness of fit with actual observations from the sample data. We used the following fit indices to evaluate the model/data fit: Chi-square, Chi-square/df, root mean square error of approximation (RMSEA), comparative fit index (CFI), and standardized root mean squared residual (SRMR). A Chi-square/df below 3, an RMSEA below 0.08 (52), CFI estimates greater than 0.9, and an SRMR below 0.08 are indicative of reasonable model-data fit (53). A combination of the full set of indices was used to evaluate the model fit. Then, using the MLR estimation procedure, we tested the mediating effects of EF on the relationship between DCDQ and SRS in the ASD-ID group and ASD-only group, respectively. A two-tailed p-value < 0.05 indicated statistical significance.

## Results

### Demographic data

All of 182 autistic children (7.61±1.31 years) were included in the final analysis, as shown in **Supplementary Table 2**. Compared with the ASD-only group, ASD-ID group received higher scores in EF (67.07±8.94 vs. 61.98±9.35, p<0.001), social cognition (20.44±4.06 vs. 15.90±5.24, p<0.001), social communication (33.16±7.63 vs. 26.22±8.86, p<0.001), social motivation (14.82±4.17 vs. 12.04±5.11, p=0.001), and autistic mannerisms (17.09±5.45 vs. 13.19±5.88, p<0.001), and had poor performance in fine motor/handwriting (9.71±4.01 vs. 11.77±3.56, p=0.001), as shown in **Table 1**.

**Table 1:** Demographic characteristics of the participants grouped by cognitive level. (n=182).

Characteristics		ASD-ID (N=45)	ASD-only (N=137)	T value/χ^2^ value	*P* value
Age (mean ± SD)		7.68±1.38	7.59±1.29	0.379	0.705
Gender	Boys	84.4%	89.1%	0.676	0.411
	Girls	15.6%	10.9%		
Right-handed	Yes	75.9%	78.3%	0.077	0.781
	No	24.1%	21.7%		
Only-child	Yes	60.0%	46.0%	2.662	0.103
	No	40.0%	54.0%		
Maternal age (mean ± SD)		36.13±4.49	37.29±3.48	-1.797	0.074
If the mother has a bachelor degree or above	Yes	77.8%	81.8%	0.344	0.557
	No	22.2%	18.2%		
Per capita family income	<8000 Yuan	73.3%	49.6%	7.703	0.006*
	≥8000 Yuan	26.7%	50.4%		
Intervention history	Yes	57.1%	68.5%	1.516	0.218
	No	42.9%	31.5%		
DCDQ (mean ± SD)					
Fine motor/handwriting		9.71±4.01	11.77±3.56	-3.254	0.001*
General coordination		23.96±5.24	23.28±5.71	0.705	0.482
Controlling during movement		18.84±5.13	18.28±5.18	0.630	0.529
**EF** (mean ± SD)		67.07±8.94	61.98±9.35	4.808	<0.001*
SRS (mean ± SD)					
Social awareness		11.58±2.90	10.79±2.55	1.740	0.084
Social cognition		20.44±4.06	15.90±5.24	5.315	<0.001*
Social communication		33.16±7.63	26.22±8.86	4.709	<0.001*
Social motivation		14.82±4.17	12.04±5.11	3.308	0.001*
Autistic mannerisms		17.09±5.45	13.19±5.88	3.928	<0.001*

*p<0.05; SD, standard deviation; ASD, autism spectrum disorder; IQ, Intelligence Quotient; DCDQ, The Developmental Coordination Disorder Questionnaire; EF, executive function; SRS, The Social Responsiveness Scale.

### Correlation analyses

Given that the SRS factors and EF scores differed between the cognitive groups (**Table 1**), we explored the intercorrelations of the manifest variables in each group using Spearman’s correlation analysis. The means, SD, and correlations of the manifest variables are presented in **Tables 2**, **3**. In the ASD-only group, the correlations between the DCDQ, EF, and SRS were significant. The subscales of DCDQ were all negatively correlated with EF and SRS (P all<0.05), except for the correlation of fine motor/handwriting and social awareness (p=0.074) and social motivation (p=0.141), and EF was positively correlated with all of the SRS subscales (P all<0.05). However, in the ASD-ID group, only the score of general coordination was correlated with EF (p=0.021), as was social motivation (p=0.036).

**Table 2:** Intercorrelations of DCDQ, EF and SRS among ASD-ID group(N=45).

Variables	mean	SD	1	2	3	4	5	6	7	8
DCDQ										
Fine motor/handwriting	9.71	4.01	1							
General coordination	23.96	5.24	0.352*	1						
Controlling during movement	18.84	5.13	0.432**	0.556**	1					
**EF**	67.07	8.94	-0.269	-0.351*	-0.183	1				
SRS										
Social awareness	11.58	2.90	-0.201	-0.095	-0.126	0.480**	1			
Social cognition	20.44	4.06	0.039	-0.266	-0.043	0.515**	0.453**	1		
Social communication	33.16	7.63	0.051	-0.148	-0.015	0.493**	0.613**	0.795**	1	
Social motivation	14.82	4.17	-0.075	-0.321*	-0.183	0.361*	0.486**	0.600**	0.715**	1
Autistic mannerisms	17.09	5.45	0.111	-0.143	0.018	0.401**	0.454**	0.597**	0.796**	0.620**

age, gender, and IQ were adjusted; **Correlation is significant at the 0.01 level (2-tailed); *Correlation is significant at the 0.05 level (2-tailed); DCDQ, The Developmental Coordination Disorder Questionnaire; EF, executive function; SRS, The Social Responsiveness Scale.

**Table 3:** Intercorrelations of DCDQ, EF and SRS among ASD-only group(N=137).

Variables	mean	SD	1	2	3	4	5	6	7	8
DCDQ										
Fine motor/handwriting	11.77	3.56	1							
General coordination	23.28	5.71	0.282**	1						
Controlling during movement	18.28	5.18	0.438**	0.502**	1					
**EF**	61.98	9.35	-0.369**	-0.425**	-0.258**	1				
SRS										
Social awareness	10.79	2.55	-0.154	-0.344**	-0.277**	0.508**	1			
Social cognition	15.90	5.24	-0.229*	-0.320**	-0.333**	0.530**	0.520**	1		
Social communication	26.22	8.86	-0.213*	-0.405**	-0.405**	0.629**	0.620**	0.766**	1	
Social motivation	12.04	5.11	-0.127	-0.318**	-0.303**	0.433**	0.351**	0.525**	0.658**	1
Autistic mannerisms	13.19	5.88	-0.249**	-0.435**	-0.333*	0.630**	0.533**	0.660**	0.804**	0.556**

age, and gender were adjusted; **Correlation is significant at the 0.01 level (2-tailed); *Correlation is significant at the 0.05 level (2-tailed); DCDQ, The Developmental Coordination Disorder Questionnaire; EF, executive function; SRS, The Social Responsiveness Scale.

### Structural model

#### Structural model without mediators

**Figure 1:**
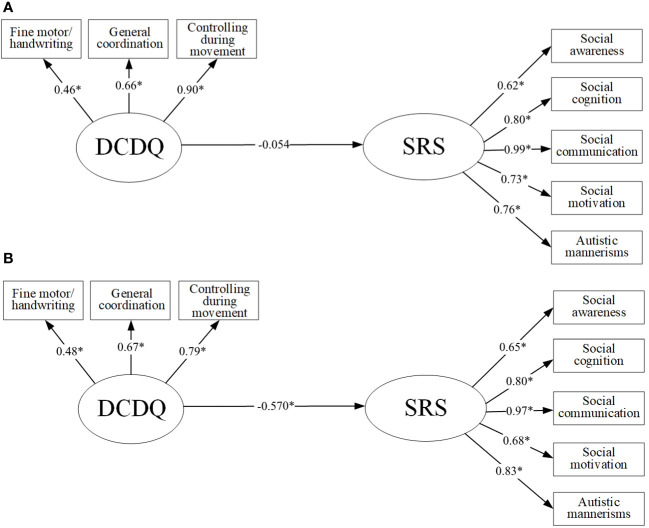
Structural equation model of the relationship between motor coordination and social communication deficit in the ASD-ID group **(A)** and ASD-only group **(B)**. All factor loadings were standardized; age, and gender were covariates for each variable; *P<0.05; DCDQ, The Developmental Coordination Disorder Questionnaire; SRS, The Social responsiveness Scale.

In the first model, the direct effects of the predictor (DCDQ) on the dependent variable (SRS) were tested without a mediator using SEM. The direct standardized path coefficients were shown in **Figure 1**. In the ASD-only group, the direct effect indicated that DCDQ was negatively significantly associated with SRS (β=-0.54, P<0.001). The obtained indices showed that the model was acceptable: Chi-square=32.784, Chi-square/df=0.993, RMSEA=0.062, CFI=1.000, and SRMR=0.061 (**Figure 1A**). In the ASD-ID group, the model fit was acceptable (Chi-square=59.050, Chi-square/df=1.789, RMSEA=0.132, CFI=0.860, SRMR=0.109), while the effect of motor coordination on social communication deficit was insignificant (p>0.05) (**Figure 1B**). **Figure 1** also depicts the unique contributions of the individual subscales (dimensions) as indicators of the global factors of DCDQ and SRS.

#### Structural model with mediators

**Figure 2:**
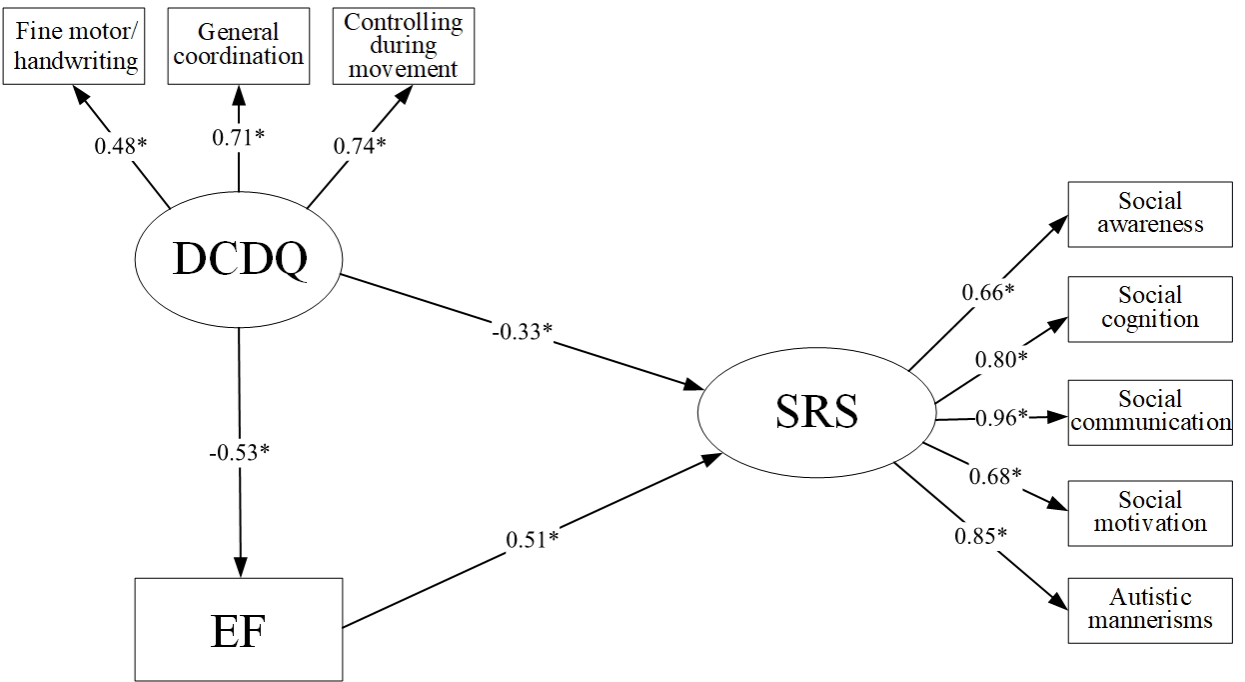
Structural equation model of the relationship between EF, motor coordination, and social communication deficit in the ASD-only group. All factor loadings were standardized; age, and gender were covariates for each variable; *P<0.05; DCDQ, The Developmental Coordination Disorder Questionnaire; SRS, The Social responsiveness Scale; EF, executive function.

Given that there was no significant direct effect of motor coordination on SRS in the ASD-ID group, we only explored the mediating effect of EF in the ASD-only group. In the mediation model, EF was included as the mediator (**Figure 2**). A significant total effect of DCDQ on SRS emerged (β=-0.599, P<0.001). When dividing the total effect into the direct effect of DCDQ and the indirect effect of EF, the direct effect of DCDQ was significant (β=-0.331, P<0.001), as was the indirect effect of EF (β=-0.268, P<0.001). This indirect effect indicated that although DCDQ had a significant correlation with SRS, EF partially mediated a negative relationship between DCDQ and SRS. The obtained indices showed that the model fit the data well (Chi-square=60.325, Chi-square/df=1.47, RMSEA=0.059, CFI=0.967, SRMR=0.069). The direct standardized path coefficients are shown in **Figure 2**. Additionally, the total effects, direct effects, and indirect effects in the mediated model with a 95% confidence interval were shown in **Table 4**.

**Table 4:** Direct, indirect and total effects for the partially mediated models of EF among ASD-only group.

Model pathways	Estimated effect	Product of Coefficients			95% CI	
		S.E.	Est./S.E.	*p*-value	Lower bonds	Upper bonds
**Total effect from DCDQ to SRS**	-0.599	0.071	-8.440	<0.001	-0.738	-0.460
Direct effect						
DCDQ → SRS	-0.331	0.085	-3.894	<0.001	-0.497	-0.164
Indirect effect						
DCDQ → EF → SRS	-0.268	0.054	-4.989	<0.001	-0.374	-0.163

All factor loadings were standardized; age, and gender were covariates for each variable.

## Discussion

This study provides an understanding of the relationship between motor coordination difficulties, executive function, and social communication deficits among autistic children. Based on a structural equation model, we confirm that motor coordination difficulties are positively associated with the severity of social communication deficits in autistic children without ID. Most importantly, when the mediating effect of EF is considered, we reveal that EF partially mediates the relationship between motor coordination difficulties and social communication deficits. To be specific, better motor coordination function may be associated with stronger EF, and the stronger EF may buffer against the social communication of autistic children.

Our findings in both models, with and without the mediator, confirm the hypothesis that improved motor coordination function is linked to a reduced social communication deficit in autistic children. This positive relationship aligns with previous cross-sectional studies (5, 6) and suggests that autistic children with better motor function may respond positively to intervention, leading to enhanced social function. In turn, the alleviation of core deficits may prompt children to engage more in physical activities and show improvements in motor function (9). However, in contrast with the findings of Ketcheson et al. (31), our research identified no significant association between motor coordination and social communication within the ASD-ID group, which might be attributed to the limited sample size in our study. In their research involving a substantial sample of 10,234 children, Ketcheson et al. revealed a strong relationship between motor coordination difficulties and social communication deficits as measured by the Social Communication Questionnaire, regardless of the presence of ID. Thus, further investigation with a larger sample size is needed to validate the relationship within the ASD-ID population.

In this research, the mediating model reveals that EF partially mediates the relationship between motor coordination difficulties and social communication deficits. Firstly, our findings are in line with previous research on the relationship between motor coordination function and EF. Motor skills have been identified as closely linked to the development of EF (54, 55), offering opportunities for interaction with the environment and other people and learning about the world (56). This is also supported by previous research suggesting that children with motor coordination difficulties had difficulties with inhibition control, working memory, and planning (57, 58). These impairments might be related to the disturbances in visuospatial processing among children with motor coordination difficulties, leading them to pay more attention to visual information while performing the tests (59). Thus, our findings showed that autistic children exhibit a similar trend. With better motor coordination function, children tended to engage more in physical activity, which provided learning experiences necessary for proper cognitive development, such as those related to paying attention, remembering instructions, and remembering the necessity to inhibit irrelevant actions (60) and further reinforced EF. Therefore, our results supported this relationship between motor coordination function and EF.

In our mediation model, the effect of EF on social communication function can be elucidated by previous studies indicating that EF dysfunction can predict social deficits in autistic children (14–17). We speculate that EF may influence social communication function by facilitating higher-order strategies, such as emotional control, initiation, and monitoring (61). Children facing emotional control challenges, such as difficulties in emotional expressions and modulating or regulating emotional responses, are prone to social rejection and isolation. Additionally, children with difficulties in initiation might find it hard to begin tasks independently or create new conversations with peers, and this consequently limited the development of their social communication (17). The intervention of EF has further underlined its role in enhancing social communication function (18). In addition, our proposal of the mediating role of EF has been supported by evidence from the neuroimaging perspective. Studies suggested that social communication deficits were associated with altered function in the frontal and parietal networks (62, 63), which were responsible for the integration of cognitive processes and executive control (63, 64).

Our mediating model of motor, EF, and social function could be partially explained by the theory of social movement synchronization (SMS) in autistic population (65, 66), which refers to synchronous motor movements within social interaction. The motor-social relationship might be attributed to the deficit of SMS among autistic children, particularly when intentional SMS tasks were involved (65). These intentional SMS tasks generally involve additional processing demands, such as attention, working memory and movement planning, posing challenges for autistic individuals with EF difficulties (67). Additionally, our mediating model of the relationship is also supported by several types of such interventions. Research showed that motor-related interventions could achieve significant effects on the improvement of social communication in autistic children (8, 36, 68–70), as well as their EF (36, 71–73). Motor-related intervention could promote the ability of action-planning and the inhibition of undesired behaviors among autistic children and provide opportunities for them to interact with peers and instructors; thus, the increased social stimulation might prompt social communication function (7). Therefore, it can be inferred that motor-related interventions have the potential to enhance the social communication of autistic children through the improvement of EF, offering a possible rationale for our results.

The results of this study have important implications in the field of autistic diagnosis and management, as motor coordination function and early life motor delay strongly predicted the diagnosis of ASD (74). Our findings add to the literature by providing a better understanding of the relationship between motor coordination difficulties, EF, and social communication deficits of autistic children and indicate the importance of motor coordination evaluation in the rehabilitation of ASD children. In addition, the potential intervention value of EF in the rehabilitation of autistic children should be considered.

The current research has several strengths. This study used a representative sample of autistic children, including children with normal cognition and ID. ASD diagnosis was made by a child psychologist through the DSM-5 criteria, and standardized scales (i.e., DCDQ, SRS, and BRIEF) were used to assess the behavior of the children.

This study suffered from several limitations. First, the motor coordination difficulties, EF, and social communication deficits were collected from parent reports; thus, self-report bias could not be excluded. In future studies, a combination of informant-report measures and objective performance-based measurements should be considered. Schilbach L et. al proposed a stimulus-response compatibility paradigm demonstrating the significant influence of gaze-mediated social context on action control (75), which could be extended to future investigations of the motor-social relationship. Furthermore, building upon the SMS theory, Schilbach L et. al introduced an unobtrusive motion tracking system (76) that allows for the quantitative measurement of social-motor relationship among autistic children in further research. Then, the cross-sectional nature of this study limited the causal claims of the relationship. Longitudinal studies with objective performance-based measurements are needed.

## Conclusion

In this research, the relationship between motor coordination function and social communication deficits of autistic children is confirmed. It indicates the importance of evaluating motor coordination in the diagnosis and management of autistic children, thus improving the prognosis of autistic children. In addition, it provides a potential mechanism explaining the association between motor coordination difficulties and social communication by having studied executive function. We conclude that executive function partially mediates the relationship between motor coordination difficulties and social communication deficits among autistic children, and both motor-related intervention and EF-related intervention should be promoted in autistic rehabilitation.

## Data availability statement

The raw data supporting the conclusions of this article will be made available by the authors, without undue reservation.

## Ethics statement

The studies involving humans were approved by Ethics Committee of Sun Yat-Sen University. The studies were conducted in accordance with the local legislation and institutional requirements. The participants provided their written informed consent to participate in this study.

## Author contributions

TG: Writing – review & editing, Writing – original draft, Software, Methodology, Investigation, Formal Analysis, Conceptualization. CJ: Writing – review & editing, Software, Methodology, Conceptualization. LL: Writing – review & editing, Supervision, Resources, Data curation. XW: Writing – review & editing, Project administration, Data curation. XL: Writing – review & editing, Project administration, Data curation. JJ: Writing – review & editing, Supervision, Resources, Methodology, Funding acquisition. MC: Writing – review & editing, Supervision, Resources, Methodology, Funding acquisition, Conceptualization.
