# Supplementary material for: The relationship between executive function and the association of motor coordination difficulties and social communication deficits in autistic children
Source: Front Psychiatry. 2024 Mar 26;15:1363406. doi: 10.3389/fpsyt.2024.1363406 (PMC11002984; doi:10.3389/fpsyt.2024.1363406)
Supplement: Supplementary file 1 [file Table_1.docx]

sTable 1. Literature review on motor coordination, social function, and executive function of autism and typically developed children

| **Author**  **(year)** | **participants** | **study setting** | **Age of sample** | **ASD diagnosis tool** | **Assessment of**  **motor function** | **Assessment of social function** | **Assessment of executive function** | **Mediators/**  **Covariables** | **If an intervention study** | **Main finding** |
| --- | --- | --- | --- | --- | --- | --- | --- | --- | --- | --- |
| Bhat et al., (2022) | ASD children from SPARK study database | USA | 5-15 years old | DSM-V,  DSM-IV | DCDQ | SCQ -lifetime version | - | Age, and gender | No | Motor impairment were positively associated with social impairment. |
| Ketcheson et al., (2021) | ASD children from SPARK study database | USA | 5-15years old | DSM-V,  DSM-IV | DCDQ | SCQ-lifetime version | - | IQ, and age | No | Motor impairments were positively associated with social impairment. |
| Cheung et al., (2021) | ASD children from SEELS data set | USA | baseline: 7-9 y; wave 2: 8-10y; wave 3: 10-12y | not mentioned | parent interview | parent interview | - | Motor function, gender, ethnicity, mother’s education level, and annual  household income | No | Motor function was positively associated with social function. |
| Gong et al., (2020) | 46 high-functioning ASD, 12 low-functioning ASD and 30 TD | China | 4-6 years old | DSM-V | Plantar pressure measurements | 1. Autism Spectrum Quotient  2.SRS | - | IQ | No | Motor impairment were positively associated with social impairment. |
| Craig et al., (2018) | 46 ASD+ID, 42 ID and 43 TD | Italy | 3-6 years old | DSM-V, ADOS-2 | MABC-2 | 1. SCQ 2. ACSF:SC | - | IQ | No | Motor impairment were positively associated with social impairment. |
| Kostrubiec et al., (2018) | 20 high-functioning ASD and 21 TD | France | 8-14 years old | DSM-IV,  ADI-R | 1. DCDQ 2. relative phase between green and red dots | 1. VABS-II 2. SCQ | - | Age | No | Motor function was positively associated with social function. |
| Kaur et al., (2018) | 24 ASD and 12 TD, from an RCT | USA | 5-12 years old | ADOS-2 | 1.Bruininks-Oseretsky Test of Motor Proficiency 2.Bilateral Motor Coordination subtest of the Sensory Integration and PraxisTest | ADOS-2 | - | - | No | Motor impairments were positively associated with severer ASD symptom. |
| Mody et al., (2017) | children with ASD enrolled in the Autism Treatment Network | USA | 2-17 years old | DSM-IV, ADOS | 1. VABS-II 2. MSEL | 1. VABS-II 2. ADOS 3. MSEL | - | Age, non-verbal IQ, ADHD medication use, and muscle tone | No | Motor function was positively associated with social function. |
| Hannant et al., (2016) | 18ASC and 18 TD | UK | 6-16y | DSM-IV,  ADI-R,  ADOS-2 | 1. MABC 2. Beery-Buktenica Developmental Test, Sixth Edition | SCQ、ADOS-2、ADI-R | - | - | No | Motor impairments were positively associated with social impairment. |
| MacDonald et al., (2013) | 35 ASD children with IQ above 64 | USA | 6-15 years old | ADOS | TGMD-2 | 1. SSIS  2. ADOS | - | Age, IQ, ethnicity, gender, and clinical ASD diagnosis | No | Motor impairments were positively associated with social impairment. |
| Travers et al., (2013) | 26 ASD and 26 TD individuals with IQ above 80 | USA | 16-28 years old | ADOS-G,  ADI-R | use Wii balance board to measure balance | SRS | - | - | No | Motor impairments were positively associated with social impairment. |
| Linkenauger et al., 2012 | E1. 12 ASD and 12 TD;  E2. 8 ASD and 8 TD | Germany | E1:9-13 y E2:17-34 y | ADOS | tasks that require perceptual–motor integration | SCQ | - | IQ | No | Motor impairments were positively associated with social impairment. |
| Bishop-Fitzpatrick et al., (2017) | 108 ASD individual participants with IQ above 70 | USA | 9-27 years old | ADOS-G,  ADI-R | Halstead-Reitan battery | VABS | - | Gender, age, and IQ | No | Social function was not associated with motor function, but with social cognition. |
| Pusponegoro et al., (2016) | 40 ASD and 40 TD matched with age | Indonesia | 18months-  6 years old | DSM-V | VABS-II | VABS-II | - | - | No | Motor function was not associated with social function. |
| Fong & Iarocci, (2020) | 77 ASD and 55 TD with IQ above 85 | Canada | 7-13 years old | DSM-IV; ADOS | - | MSCS | BRIEF | Maternal education and child gender | No | Executive function was positively associated with social function. |
| Bednarz et al., (2020) | 106 ASD from ABIDE-II database | USA | 5-13 years old | ADI-R,  ADOS-2 | - | SRS | BRIEF | Age, and IQ | No | Executive function was positively associated with social function. |
| Chouinard et al., (2019) | 146 ASD from ABIDE-II database, with IQ above 85 | USA | 5-18 years old | Not mentioned | - | SRS | BRIEF | Gender, age and IQ | No | Executive function was positively associated with social function. |
| Torske et al., (2017) | 86 ASD with IQ above 70 | Norwegian | 6-18 years old | ICD-10, ADOS, ADI-R, SCQ | - | SRS | BRIEF | Gender, age and IQ | No | Executive function (Metacognition Index) was positively associated with social function. |
| Leung et al., (2016) | 70 ASD and 71 TD | UK | 6-15 years old | ADOS | - | SRS | BRIEF | Gender, and age | No | Executive function was positively associated with social function. |
| Wilson et al., (2020) | 52 DCD and 134 TD | Australia | 6-11 years old | Not ASD | MAND | - | GMLT | - | No | Children with DCD have significantly worse executive function than TD. |
| Sartori et al., (2020) | 63 DCD, 31 r-DCD and 63 TD | Brazil | 8-9 years old | Not ASD | MABC-2 | - | 1. Oral word span in sentences; Odd-One-Out  2. GNG; Hayling test  3. TMT; FDT | - | No | Children with DCD have significantly worse executive function than TD. |
| Ke et al., (2019) | 75 DCD, 35 r-DCD and 391 TD | China | 7-10 years old | Not ASD | MABC-2 | - | impulse control and information-updating tasks | Gender, age, parental years of education, and family income | No | Motor function was positively associated with executive function. |
| Bernardi et al., (2018) | 17 DCD, 17 r-DCD and 17 TD | UK | 7-11 years old | Not ASD | MABC-2 | - | executive function assessment battery | Age | No | Children with DCD have significantly worse executive function than TD. |
| Zhao & Chen, (2018) | 41 ASD from a special school | China | 5-8 years old | DSM-V | - | 1. SSIS  2. ABLLS-R | - | - | Yes (Structural physical activity program) | Physical intervention positively influenced social function. |
| Caputo et al., (2018) | 26 children with ASD | Italy | 6-12 years old | DSM-V, ADOS,  ADI-R | - | 1. CARS  2. VABS | - | - | Yes  (Multisystem Aquatic Therapy) | Multisystem aquatic therapy positively influenced social function. |
| Borgi et al., (2016) | 28 ASD with IQ above 70 | Italy | 6-12 years old | DSM-IV-TR,  ICD-10 | - | VABS | TOL | - | Yes  (Equine-assisted therapy) | Equine-assisted therapy positively influenced social function, and executive function. |
| Bahrami et al., (2016) | 30 children with ASD | Iran | 5-16 years old | DSM-IV-TR | - | GARS-2 | - | - | Yes  (Karate Techniques Training) | Karate Techniques Training positively influenced social function. |
| Bass et al., (2009) | 34 children with ASD | USA | 5-10 years old | DSM-IV-TR | - | SRS | - | - | Yes  (Therapeutic Horseback Riding) | Therapeutic Horseback Riding positively influenced social function. |
| Kenworthy et al., (2014) | 67 ASD | USA | 7-11 years old | ADOS; DSM-IV-TR | - | SRS | BRIEF | - | Yes  (Unstuck and On Target) | EF intervention positively influenced social function. |
| Tse et al., (2021) | 62 children with ASD | China (Hongkong) | 8-12 years old | DSM-V, ADOS-2 | - | - | 1.TOL;  2. CBTT, FDS, BDS,  3. SCWT;  4.GNG | Age, and IQ | Yes  (Learning to bicycle) | Motor intervention positively influenced EF. |
| Tse et al., (2019) | 40 children with ASD | China (Hongkong) | 8-12 years old | DSM-V, ADOS-2 | - | - | 1. GNG  2. CBTT; FDS; BDS | Age | Yes  (Basketball skill learning intervention) | Motor intervention positively influenced EF. |
| Pan et al., (2017) | 22 children with ASD | China (Taiwan) | 6-12 years old | DSM-IV-TR | Bruininks-Oseretsky Test of Motor Proficiency | - | Wisconsin Card Sorting Test | Age | Yes  (Physical activity intervention) | Motor intervention positively influenced EF. |

**Abbreviations:** ASD, autism spectrum disorder; TD, typically developmental; ID, intellectual disability; DSM, Diagnostic and Statistical Manual of Mental Disorders; ADOS, the Autism Diagnostic Observation Schedule; PEP-3, Psychoeducational Profile, Third Edition; ADI-R, Autism Diagnostic Interview Revised; M-CHAT, the Modified Checklist for Autism in Toddlers; DCDQ, Developmental Coordination Disorder Questionnaire; SCQ, The Social Communication Questionnaire; ACSF:SC, the Autism Classification System of Functioning: Social Communication; SSRS, the Social Skills Rating Systems; SRS, the Social Responsiveness Scale; SSIS, Social Skills Improvement System Rating Scales; ABLLS-R, the Assessment of Basic Language and Learning Skills-Revised; VABS, Vineland Adaptive Behavior Scale; MSEL, Mullen Scales of Early Learning; TGMD, Test of Gross Motor Development; TOL, Tower of London; GARS-2, Gilliam Autism Rating Scale-Second Edition; BRIEF, Behavior Rating Inventory of Executive Function; MAND, McCarron Assessment of Neuromuscular Development; GMLT, Groton Maze Learning Test; GNG, Go/No-go task; TMT, Trail Making Test; FDT, Five Digits Test; CBTT, Corsi block tapping task; FDS, forward digit span; BDS, backward digit span; SCWT, Stroop Color and Word Test.

**Reference:**

Bahrami, F., Movahedi, A., Marandi, S. M., & Sorensen, C. (2016). The Effect of Karate Techniques Training on Communication Deficit of Children with Autism Spectrum Disorders. *J Autism Dev Disord*, *46*(3), 978-986. https://doi.org/10.1007/s10803-015-2643-y

Bass, M. M., Duchowny, C. A., & Llabre, M. M. (2009). The effect of therapeutic horseback riding on social functioning in children with autism. *J Autism Dev Disord*, *39*(9), 1261-1267. https://doi.org/10.1007/s10803-009-0734-3

Bednarz, H. M., Trapani, J. A., & Kana, R. K. (2020). Metacognition and behavioral regulation predict distinct aspects of social functioning in autism spectrum disorder. *Child Neuropsychol*, *26*(7), 953-981. https://doi.org/10.1080/09297049.2020.1745166

Bernardi, M., Leonard, H. C., Hill, E. L., Botting, N., & Henry, L. A. (2018). Executive functions in children with developmental coordination disorder: a 2-year follow-up study. *Developmental Medicine & Child Neurology*, *60*(3), 306-313. https://doi.org/10.1111/dmcn.13640

Bhat, A. N., Boulton, A. J., & Tulsky, D. S. (2022). A further study of relations between motor impairment and social communication, cognitive, language, functional impairments, and repetitive behavior severity in children with ASD using the SPARK study dataset. *Autism Res*. https://doi.org/10.1002/aur.2711

Bishop-Fitzpatrick, L., Mazefsky, C. A., Eack, S. M., & Minshew, N. J. (2017). Correlates of Social Functioning in Autism Spectrum Disorder: The Role of Social Cognition. *Res Autism Spectr Disord*, *35*, 25-34. https://doi.org/10.1016/j.rasd.2016.11.013

Borgi, M., Loliva, D., Cerino, S., Chiarotti, F., Venerosi, A., Bramini, M., . . . Cirulli, F. (2016). Effectiveness of a Standardized Equine-Assisted Therapy Program for Children with Autism Spectrum Disorder. *J Autism Dev Disord*, *46*(1), 1-9. https://doi.org/10.1007/s10803-015-2530-6

Caputo, G., Ippolito, G., Mazzotta, M., Sentenza, L., Muzio, M. R., Salzano, S., & Conson, M. (2018). Effectiveness of a Multisystem Aquatic Therapy for Children with Autism Spectrum Disorders. *J Autism Dev Disord*, *48*(6), 1945-1956. https://doi.org/10.1007/s10803-017-3456-y

Cheung, W. C., Meadan, H., & Xia, Y. (2021). A Longitudinal Analysis of the Relationships Between Social, Communication, and Motor Skills Among Students with Autism. *J Autism Dev Disord*. https://doi.org/10.1007/s10803-021-05328-7

Chouinard, B., Gallagher, L., & Kelly, C. (2019). He said, she said: Autism spectrum diagnosis and gender differentially affect relationships between executive functions and social communication. *Autism*, *23*(7), 1793-1804. https://doi.org/10.1177/1362361318815639

Craig, F., Lorenzo, A., Lucarelli, E., Russo, L., Fanizza, I., & Trabacca, A. (2018). Motor competency and social communication skills in preschool children with autism spectrum disorder. *Autism Res*, *11*(6), 893-902. https://doi.org/10.1002/aur.1939

Fong, V. C., & Iarocci, G. (2020). The Role of Executive Functioning in Predicting Social Competence in Children with and without Autism Spectrum Disorder. *Autism Res*, *13*(11), 1856-1866. https://doi.org/10.1002/aur.2350

Gong, L., Liu, Y., Yi, L., Fang, J., Yang, Y., & Wei, K. (2020). Abnormal Gait Patterns in Autism Spectrum Disorder and Their Correlations with Social Impairments. *Autism Res*, *13*(7), 1215-1226. https://doi.org/10.1002/aur.2302

Hannant, P., Cassidy, S., Tavassoli, T., & Mann, F. (2016). Sensorimotor Difficulties Are Associated with the Severity of Autism Spectrum Conditions. *Front Integr Neurosci*, *10*, 28. https://doi.org/10.3389/fnint.2016.00028

Kaur, M., S, M. S., & A, N. B. (2018). Comparing motor performance, praxis, coordination, and interpersonal synchrony between children with and without Autism Spectrum Disorder (ASD). *Res Dev Disabil*, *72*, 79-95. https://doi.org/10.1016/j.ridd.2017.10.025

Ke, L., Duan, W., Xue, Y., & Wang, Y. (2019). Developmental Coordination Disorder in Chinese Children Is Correlated With Cognitive Deficits. *Frontiers in Psychiatry*, *10*. https://doi.org/10.3389/fpsyt.2019.00404

Kenworthy, L., Anthony, L. G., Naiman, D. Q., Cannon, L., Wills, M. C., Luong-Tran, C., . . . Wallace, G. L. (2014). Randomized controlled effectiveness trial of executive function intervention for children on the autism spectrum. *J Child Psychol Psychiatry*, *55*(4), 374-383. https://doi.org/10.1111/jcpp.12161

Ketcheson, L. R., Pitchford, E. A., & Wentz, C. F. (2021). The Relationship Between Developmental Coordination Disorder and Concurrent Deficits in Social Communication and Repetitive Behaviors Among Children with Autism Spectrum Disorder. *Autism Res*, *14*(4), 804-816. https://doi.org/10.1002/aur.2469

Kostrubiec, V., Huys, R., Jas, B., & Kruck, J. (2018). Age-dependent Relationship Between Socio-adaptability and Motor Coordination in High Functioning Children with Autism Spectrum Disorder. *J Autism Dev Disord*, *48*(1), 209-224. https://doi.org/10.1007/s10803-017-3326-7

Leung, R. C., Vogan, V. M., Powell, T. L., Anagnostou, E., & Taylor, M. J. (2016). The role of executive functions in social impairment in Autism Spectrum Disorder. *Child Neuropsychol*, *22*(3), 336-344. https://doi.org/10.1080/09297049.2015.1005066

Linkenauger, S. A., Lerner, M. D., Ramenzoni, V. C., & Proffitt, D. R. (2012). A perceptual-motor deficit predicts social and communicative impairments in individuals with autism spectrum disorders. *Autism Res*, *5*(5), 352-362. https://doi.org/10.1002/aur.1248

MacDonald, M., Lord, C., & Ulrich, D. A. (2013). The relationship of motor skills and social communicative skills in school-aged children with autism spectrum disorder. *Adapt Phys Activ Q*, *30*(3), 271-282. https://doi.org/10.1123/apaq.30.3.271

Mody, M., Shui, A. M., Nowinski, L. A., Golas, S. B., Ferrone, C., O'Rourke, J. A., & McDougle, C. J. (2017). Communication Deficits and the Motor System: Exploring Patterns of Associations in Autism Spectrum Disorder (ASD). *J Autism Dev Disord*, *47*(1), 155-162. https://doi.org/10.1007/s10803-016-2934-y

Pan, C. Y., Chu, C. H., Tsai, C. L., Sung, M. C., Huang, C. Y., & Ma, W. Y. (2017). The impacts of physical activity intervention on physical and cognitive outcomes in children with autism spectrum disorder. *Autism*, *21*(2), 190-202. https://doi.org/10.1177/1362361316633562

Pusponegoro, H. D., Efar, P., Soedjatmiko, Soebadi, A., Firmansyah, A., Chen, H. J., & Hung, K. L. (2016). Gross Motor Profile and Its Association with Socialization Skills in Children with Autism Spectrum Disorders. *Pediatr Neonatol*, *57*(6), 501-507. https://doi.org/10.1016/j.pedneo.2016.02.004

Sartori, R. F., Valentini, N. C., & Fonseca, R. P. (2020). Executive function in children with and without developmental coordination disorder: A comparative study. *Child: Care, Health and Development*, *46*(3), 294-302. https://doi.org/10.1111/cch.12734

Torske, T., Naerland, T., Oie, M. G., Stenberg, N., & Andreassen, O. A. (2017). Metacognitive Aspects of Executive Function Are Highly Associated with Social Functioning on Parent-Rated Measures in Children with Autism Spectrum Disorder. *Front Behav Neurosci*, *11*, 258. https://doi.org/10.3389/fnbeh.2017.00258

Travers, B. G., Powell, P. S., Klinger, L. G., & Klinger, M. R. (2013). Motor difficulties in autism spectrum disorder: linking symptom severity and postural stability. *J Autism Dev Disord*, *43*(7), 1568-1583. https://doi.org/10.1007/s10803-012-1702-x

Tse, A. C. Y., Anderson, D. I., Liu, V. H. L., & Tsui, S. S. L. (2021). Improving Executive Function of Children with Autism Spectrum Disorder through Cycling Skill Acquisition. *Med Sci Sports Exerc*, *53*(7), 1417-1424. https://doi.org/10.1249/mss.0000000000002609

Tse, C. Y. A., Lee, H. P., Chan, K. S. K., Edgar, V. B., Wilkinson-Smith, A., & Lai, W. H. E. (2019). Examining the impact of physical activity on sleep quality and executive functions in children with autism spectrum disorder: A randomized controlled trial. *Autism*, *23*(7), 1699-1710. https://doi.org/10.1177/1362361318823910

Wilson, P., Ruddock, S., Rahimi‐Golkhandan, S., Piek, J., Sugden, D., Green, D., & Steenbergen, B. (2020). Cognitive and motor function in developmental coordination disorder. *Developmental Medicine & Child Neurology*, *62*(11), 1317-1323. https://doi.org/10.1111/dmcn.14646

Zhao, M., & Chen, S. (2018). The Effects of Structured Physical Activity Program on Social Interaction and Communication for Children with Autism. *Biomed Res Int*, *2018*, 1825046. https://doi.org/10.1155/2018/1825046
