# Supplementary material for: The relationship between executive function and the association of motor coordination difficulties and social communication deficits in autistic children
Source: Front Psychiatry. 2024 Mar 26;15:1363406. doi: 10.3389/fpsyt.2024.1363406 (PMC11002984; doi:10.3389/fpsyt.2024.1363406)
Supplement: Supplementary file 2 [file Table_2.docx]

sTable 2. Demographic characteristics of the participants. (n=182)

| Characteristics |  | percentage |
| --- | --- | --- |
| Age (mean ± SD) |  | 7.61±1.31 |
| Gender | Boys | 87.9% |
|  | Girls | 12.1% |
| Only-child | Yes | 49.5% |
|  | No | 50.5% |
| Right Handedness | Yes | 77.8% |
|  | No | 22.2% |
| Maternal age (mean ± SD) |  | 37.01±3.78 |
| If the mother has a bachelor degree or above | Yes | 80.8% |
|  | No | 19.2% |
| Per capita family income | <8000 Yuan | 55.5% |
|  | ≥8000 Yuan | 44.5% |
| IQ | < 70 | 24.7% |
|  | ≥ 70 | 75.3% |
| Intervention history | Yes | 65.8% |
|  | No | 34.2% |

SD, standard deviation; ASD, autism spectrum disorder; IQ, Intelligence Quotient.
